# The viability of lytic bacteriophage ΦD5 in potato-associated environments and its effect on *Dickeya solani* in potato (*Solanum tuberosum* L.) plants

**DOI:** 10.1371/journal.pone.0183200

**Published:** 2017-08-11

**Authors:** Robert Czajkowski, Anna Smolarska, Zofia Ozymko

**Affiliations:** Department of Biotechnology, Intercollegiate Faculty of Biotechnology, University of Gdansk and Medical University of Gdansk, Gdansk, Poland; Dong-A University, REPUBLIC OF KOREA

## Abstract

*Dickeya solani* is one of the most important pectinolytic phytopathogens responsible for high losses in potato, especially in seed potato production in Europe. Lytic bacteriophages can affect the structure of the host population and may influence spread, survival and virulence of the pathogen and in consequence, infection of the plant. In this study, we aimed to acquire information on the viability of the broad host lytic bacteriophage ΦD5 on potato, as well as to apprehend the specific effect of this bacteriophage on its host *D*. *solani* type-strain in different settings, as a preliminary step to target co-adaptation of phages and host bacteria in plant environment. Viability of the ΦD5 phage in tuber extract, on tuber surface, in potting compost, in rainwater and on the leaf surface, as well as the effect of copper sulfate, were examined under laboratory conditions. Also, the interaction of ΦD5 with the target host *D*. *solani in vitro* and in compost-grown potato plants was evaluated. ΦD5 remained infectious in potato tuber extract and rain water for up to 72 h but was inactivated in solutions containing 50 mM of copper. The phage population was stable for up to 28 days on potato tuber surface and in potting compost. In both, tissue culture and compost-grown potato plants, ΦD5 reduced infection by *D*. *solani* by more than 50%. The implications of these findings are discussed.

## Introduction

Potato blackleg and tuber soft rot caused by soft rot *Enterobacteriaceae* (SRE), *Pectobacterium* spp. and *Dickeya* spp., result in important economic losses in crop production in Europe and worldwide [1]. The significance of *Dickeya* spp. (former *Erwinia chrysanthemi* and *Pectobacterium chrysanthemi*) as a potato pathogen has been increasing in the past ten years [1–4]. This has been associated mainly with the introduction and spread of a new *Dickeya* species named *D*. *solani* [5]. The presence of this species has been reported in many European countries, as well as in Brazil, Turkey, Israel and Georgia [1].

Control of the SRE bacteria and specifically *D*. *solani* in potato is hampered by the lack of efficient measures against these pathogens [6]. Until now, as the disease is primarily seed-borne, the focus has been on an integrated strategy including the use of healthy certified seed potato material derived from pathogen-free minitubers and/or stem cuttings, hygienic practices during planting, cultivation and storage, avoiding of tubers’ wounding and effective drainage of fields. Nevertheless, even then, only partial control of the disease has been achieved. This can be attributed to failure in achieving a consistent reduction of the bacterial inoculum level on the seed tubers and hence infection incidences under field conditions [6].

As an addition to the above-mentioned conventional methods of potato disease control [6], the use of SRE-specific lytic bacteriophages has been considered [7]. Bacteriophages are viruses able to infect and kill bacterial cells. They are self-replicating, may persist in the environment for longer periods, are safe to use, as they are non-infectious to humans, animals and/or plants [8]. It has been repeatedly reported that bacteriophages have the potential to control plant pathogenic bacteria. For example, the use of lytic phages in controlling *Erwinia amylovora* (fire blight) in apple and pear, *Xanthomonas pruni* (bacterial spot of peach) in peach, *Xanthomonas campestris* pv. *pelargonii* (bacterial blight of geranium) in geranium, *Xanthomonas campestris* pv. *vesicatoria* (bacterial spot of tomato) in tomato, *Pseudomonas tolaasii* (brown blotch) in mushrooms and against *Ralstonia solanacearum* (potato brown rot) and *Streptomyces scabies* (potato scab) in potato was described earlier [9].

Several bacteriophages have been isolated against *Pectobacterium* spp. and *Dickeya* spp. with reference to studies on identification and taxonomy of the bacterial hosts [10]. However, as the ecology of SRE-infecting bacteriophages is largely unknown, their practical application is limited. For example, little has been demonstrated on the survival of SRE-associated lytic bacteriophages in soil and on/in potato tubers and on their interaction with the host bacteria in environment [10]. The understanding of interactions between bacteria and bacteriophages remains crucial for realizing the impact of bacterial viruses on the SRE populations. It is also important for the development of an effective biological control approach based on lytic bacteriophages that could be applied under field conditions [11].

Recently, we have isolated and described a broad host lytic bacteriophage ΦD5 capable of infecting strains that belong to four potato-associated *Dickeya* species (*D*. *solani*, *D*. *dianthicola*, *D*. *dadantii* and *D*. *zeae*) [12, 13]. In the proof-of-concept assays with host bacteria, ΦD5 was able to stop the growth and efficiently lyse *D*. *solani* IPO2222, *D*. *dianthicola* IFB0103, *D*. *dadantii* 3937 and *D*. *zeae* IFB 0110 cells as it was visualized by transmission electron microscopy [13].

The bacteriophage ΦD5 has been isolated from arable soil in Poland and characterized in detail in our laboratory for features which are potentially involved in its *in vitro* viability and lytic activity against *Dickeya* spp. Transmission electron microscopy revealed that the ΦD5 phage has an icosahedral head and contractive tail and therefore was classified as a member of the family *Myoviridae*, order *Caudovirales*. Basing on the ΦD5 annotated genome sequence and the capsid morphology, the bacteriophage was classified as a member of *Viunalikevirus* genus, which includes the LIMEstone1 type isolate [14, 15].

The core of LIMEstone1 and ΦD5 genomes consists of 182 genes (more than 90% of total genes), which suggest that ΦD5 is a very close relative of the LIMEstone1 bacteriophage. This close relationship was rather unexpected as the two bacteriophages have been isolated in Belgium and Poland, respectively [13–15]. Despite the fact that both phages share large core genome and some of morphological features (e.g. capsid shape and size), they differ in host range. It cannot be excluded that the 14 unique genes present in ΦD5 and absent in LIMEstone1 genome, may be responsible for the broader host range observed in case of ΦD5 [12].

Our objective in this study was to further explore the viability/survival of ΦD5 under different environmental conditions (in compost and potato tubers). Moreover, the effect of ΦD5 presence on *D*. *solani* strain IPO2254 (type strain of *D*. *solani* (IPO2222) tagged with green fluorescent protein (GFP)) infecting potato plants was examined. For that purpose, axenic potato plants grown in culture tubes and plants potted in non-sterile potting compost in growth chambers were used in the experiments, as a preliminary step, to assess the interaction of ΦD5 and the host bacteria, as suggested previously [16, 17].

## Materials and methods

### Bacterial strains, bacteriophages and media used

GFP-tagged *D*. *solani* IPO2222 (IPO2254) [2, 18] was grown at 28°C for 24–48 h on tryptone soya agar (TSA) (Oxoid) supplemented with 150 μg ml^-1^ ampicillin (Sigma). Liquid IPO2254 cultures were prepared in nutrient broth (NB) (Oxoid) and/or in tryptic soya broth (TSB) (Oxoid), if required—also supplemented with ampicillin and grown at 28°C for 24 h with agitation (200 rpm). The GFP-tagged strain was used in all experiments instead of untagged wild type *D*. *solani* IPO2222 to improve detection and to reduce the possible bacterial background on Petri dishes with plated plant extracts as suggested previously [19]. The IPO2254 is otherwise indistinguishable from the wild type IPO2222, as it was shown in our former studies [18].

Stock suspensions of bacteriophage ΦD5, isolated and characterized in our previous studies [12, 13], were prepared by propagation on *D*. *solani* IPO2254 cultures as described previously [13]. Before each experiment the phage particles were freshly tittered and, when necessary, enriched in the IPO2254 culture to obtain the desired density of plaque forming units (pfu) ml^-1^ using soft top agar method [19]. For each experiment, the freshly tittered ΦD5 was serially diluted in the test medium (e.g. potato tuber extract, rain water, CuSO_4_ solution in sterile water or Ringer’s buffer) to obtain the desired final pfu ml^-1^.

### Determination of effect of CuSO_4_, potato tuber extract and rain water on ΦD5 fitness *in vitro*

#### Effect of potato tuber extract

Potato tuber extract was prepared as previously described [20]. Briefly, ware (table) potato tubers of cv. Bryza obtained locally in Gdansk, Poland, were washed using running tap water, surface-sterilized with 70% ethanol and 1% sodium hypochlorite, peeled and ground for 2–4 min in a food processor (Braun), after adding twice the weight of 1/4 Ringer’s buffer (Merck) containing 0.02% diethyldithiocarbamic acid (DIECA) (Sigma) as an antioxidant (Perombelon & van Der Wolf, 2002). The freshly prepared tuber extract was tested for absence of putative bacteriophages in a soft top agar assay as described above. Likewise, the potato tuber extracts were tested for presence of pectinolytic bacteria by plating in tetraplicates 100 μl of undiluted fresh tuber extract on crystal violet pectate (CVP) medium as previously described [18].

The effect of potato extract on bacteriophage survival was tested in potato tuber extracts diluted with Ringer’s buffer 1 and 10 times, at 22°C by adding ΦD5 suspensions adjusted to a final concentration of 10^3^ plaque forming units (pfu) ml^-1^ tuber extract. As a control Ringer’s buffer was used. Flasks were incubated for 72 h at 22°C and 100 μl samples in duplicates were collected at 0, 24, 48 and 72 hours and assayed for the phage presence by the soft top agar method as described above.

#### Effect of rainwater

Rainwater was collected from April to September 2015 in the region of Gdansk, northern Poland. (According to the Polish law, for rain water collection, no special permissions granted by the local and national authorities are required). Each time half of the collected water was filter sterilized with 0.22 μm syringe filter (active cellulose filter, VWR) and half left unsterilized. Both were stored at 4°C until required. The pH of the water samples was determined and the presence of putative bacteriophages assessed as described above. ΦD5 suspensions in sterile water (stock) were adjusted to a final concentration of 10^3^ plaque forming units (pfu) ml^-1^ in rain water. Flasks with bacteriophages were incubated for 72 h at room temperature (ca. 22°C). 100 μl samples in duplicates were collected at 0, 24, 48 and 72 hours and assayed for the phage presence by the soft top agar method. As a control, sterile demineralized water was used.

#### Effect of CuSO_4_

Bacteriophage viability in 0.05, 0.25, 0.5, 5 and 50 mM CuSO_4_ (Sigma) solutions in demineralized sterile water was tested at 22°C. ΦD5 suspensions were adjusted to a final concentration of 10^3^ plaque forming units (pfu) ml^-1^. Flasks with bacteriophages were incubated for 72 h. Two duplicated samples of 100 μl samples were collected at 0, 24, 48 and 72 hours and assayed for the phage presence by the soft top agar method described above. As a control, sterilized demineralized water was used.

#### Determination of viability of the ΦD5 bacteriophage on the surface of potato tubers

Potato tubers of cv. Bryza obtained as mentioned earlier were washed under running tap water and dried under laminar air flow prior to the experiments. Ten randomly selected tubers were assayed beforehand for putative phage presence. To investigate the viability of ΦD5 on tuber surface, phage concentration was adjusted to 10^3^ pfu ml^-1^ in sterile Ringer’s buffer. Ten ml of phage suspension was sprayed on the surface of each tuber. As a control, sterile Ringer’s buffer alone was used. The tuber samples were dried under the air flow for 1 h and subsequently were kept at 4–6°C and 80% RH in the dark and assayed for phage presence at 0, 7, 14, 21 and 28 days. At each time point, 10 randomly selected tubers were collected, separately weighted and washed twice in 10 ml of sterile Ringer’s buffer (Merck) (total washing volume per tuber: 20 ml) for 10 min by gently shaking at 50 rpm at room temperature to rinse off phage particles from the tuber surface. Per each sample, the two washings were combined and were filter-sterilized, serially-diluted in Ringer’s buffer and assayed for the phage presence by the soft top agar method described previously.

#### Determination of the viability of the ΦD5 bacteriophage in sterilized and unsterilized potting compost

The viability of ΦD5 was tested in unsterilized and sterilized potting compost (COMPO SANA^®^ Universal Potting Compost, Compo) at 10°C under laboratory conditions. The potting compost was sterilized by dry-heating to 160°C for 4 h, cooled to room temperature and kept under sterile conditions for the course of the experiment. Unsterilized potting compost was checked beforehand for the presence of putative bacteriophages in a soft top agar assay as described above. Sterilized and unsterilized potting compost was adjusted to 50% field capacity with demineralized sterile water. 100 g of sterilized and unsterilized compost at 50% field capacity in five repetitions each was spiked with 10 ml of 10^6^ pfu ml^-1^ of ΦD5 and mixed to assure uniform distribution of phage particles in soil. As a control, sterilized and unsterilized compost samples without bacteriophage supplementation were used. Compost samples were collected at 0, 7, 14, 21 and 28 days and assayed for the phage presence. At each time point, 1 gram of compost was randomly collected from each pot (five repetitions) and separately suspended in 10 ml of 1/4 Ringer’s buffer (Merck) supplemented with 0.02% diethyldithiocarbamic acid (Sigma). The test compost samples were shaken for 24 h at 100 rpm, centrifuged (10 min 6000 × *g*) to remove compost particles, the resulting supernatant filter was sterilized with 0.22 μm filter (VWR) before serial dilutions in Ringer’s buffer, and assayed for the phage presence by the soft top agar method.

#### Determination of viability of the ΦD5 bacteriophage on the surface of detached potato leaves

Ware (table) potato tubers of cv. Bryza, obtained as mentioned above, were stored at 4°C and 80% relative humidity in the dark until the development of sprouts (ca. 2–3 months). To avoid the risks of leaf contamination by tuber-borne bacteria, white-yellowish sprouts of a length of ca. 5–10 cm were carefully removed from tubers, planted in water-soaked peat pellets (Jiffy, Vilmorin) and placed in a humid box in the growth chamber at 26°C under white fluorescent light with a 16 h photoperiod (white cool fluorescent light, Philips, TLD 58 W/84o, 30–35 μmol m^-2^ s^-1^) for rooting and shoot development. After two weeks, the rooted, green plants were transferred to 3 L pots containing potting compost (COMPO SANA^®^ Universal Potting Compost, Compo) and grown for another 2 weeks under similar conditions. Young potato leaves (third to sixth from the tip of the shoot) were detached from the plants. Phage concentration was adjusted to 10^3^ pfu ml^-1^ in sterile Ringer’s buffer. Five ml of phage suspension was sprayed on the adaxial surface of each leaf. As a control, sterile Ringer’s buffer was used. The inoculated leaves were dried under the air flow for 30 min and after that were placed, adaxial side up, on 0.5% water agar (Oxoid) in square plastic Petri dishes (100 mm x 100 mm) (Sarstedt). Up to five randomly chosen leaves were placed in each Petri dish. The leaves were kept under growth chamber conditions as described above. Leaf samples were collected at 0, 7, 14, 21 and 28 days and assayed for phage presence. At each time point, 5 randomly chosen leaves were collected from 5 randomly chosen Petri dishes and the area of each collected leaf was measured using the Easy Leaf Area software (Department of Plant Science, University of California, http://www.plant-image-analysis.org/software/easy-leaf-area). The measured leaves were separately shaken in 5 ml of Ringer’s buffer at 50 rpm at room temperature for 10 min to rinse off phage particles from the leaf surface. The washings were then serially-diluted in Ringer’s buffer and assayed for the phage presence by the soft top agar method described above. The experiment was repeated one time with the same setup and the results were averaged.

### Effect of ΦD5 bacteriophage on *D*. *solani* IPO2254 in potato plants grown in tissue cultures

#### Growth of *in vitro* potato plants and inoculation of the plants with *D*. *solani* IPO2254 and ΦD5 bacteriophage

*In vitro*-grown potato plantlets of cv. Kondor were obtained from the Seed Production and Potato Protection, Plant Breeding Acclimatization Institute—National Research Institute, Bonin, Poland. Plants were grown and propagated in culture tubes as described earlier [21].

Potato plants were inoculated with 10 μl of 10^10^ pfu ml^-1^ suspension of ΦD5 in sterile demineralized water with a pipette tip inserted into the interspace between the stem base and MS medium of each individual plant in order to avoid wounding of the stems. As a negative control, 10 μl of sterile demineralized water was used. Inoculated plants were grown at 20–22°C as described above. 24 h after the inoculation, the plants were inoculated as above with 10 μl suspension of *D*. *solani* IPO2254 in sterile water containing 10^6^ cfu ml^-1^ at the same inoculation site using a pipette tip inserted into the interspace between the stem base and MS medium of each individual plant. As a control, 10 μl of sterile demineralized water was used. Four treatments were applied: treatment A—plants inoculated with sterile water (control), treatment B—plants inoculated with ΦD5 bacteriophage, treatment C—plants inoculated with ΦD5 bacteriophage and *D*. *solani* IPO2254 and treatment D—plants inoculated with *D*. *solani* IPO2254 only. Each treatment consisted of 20 plants grown in individual culture tubes and the experiment was repeated independently one time with the same setup.

#### Symptoms development and sampling of culture-grown potato plants

Inoculated plants were visually inspected after 2, 5 and 10 days post inoculation (dpi) for wilting, typical blackleg symptoms, stem desiccation and/or plant death, and after 14 dpi they were sampled. For testing, the plants were aseptically removed from the culture tubes, 1 cm long stem fragments were collected and analyzed for the presence of *D*. *solani* IPO2254 by plating plant extracts prepared in 1/4 Ringer’s buffer on TSA and CVP supplemented with 150 μg ml^-1^ ampicillin and counting GFP-positive, ampicillin resistant bacterial colonies as described earlier [21].

### Effect of ΦD5 on *D*. *solani* IPO2254 in potted potato plants grown in a growth chamber

#### Plant growth in potting compost inoculated with GFP-tagged *D*. *solani* IPO2254 and ΦD5 bacteriophage

Duplicated experiments were performed in April and May 2015. Ware (table) potato tubers of cv. Bryza were stored at 4°C and 80% relative humidity in the dark until sprouting (ca. 1–2 months). 5–10 cm long sprouts were planted in water-soaked peat pellets (Jiffy, Vilmorin) in a damp box in a growth chamber at 26°C under white fluorescent light with a 16 h photoperiod for rooting and haulm development. After two weeks, the rooted, green plants were transferred to 3 L pots and grown in potting compost for another 2 weeks under similar conditions. Plants were watered 1 h before inoculation of compost with bacteriophages. Inoculation was carried out by immersing the pots one third down for 1 h in a suspension of ΦD5 in sterile demineralized water containing 10^8^ pfu ml^-1^ or in bacteriophage-free sterile water (negative control). Following the inoculation, the plants were left unwatered for 24 h and then watered daily to maintain the roots moist. One week after inoculation with bacteriophages, the potting mixture was inoculated with 10^6^ cfu ml^-1^ *D*. *solani* IPO2254 also by immersion of the one third down of the pots in bacterial suspension. As control, sterile demineralized water was used (double negative control). As previously, four treatments were applied, each consisting of 15 plants: treatment A1—plants inoculated with sterile water (control), treatment B1- plants inoculated with ΦD5 bacteriophage, treatment C1—plants inoculated with ΦD5 bacteriophage and *D*. *solani* IPO2254 and treatment D1—plants inoculated with *D*. *solani* strain IPO2254 only. A random plot design of the layout of pots was applied: 3 blocks of 5 pots per treatment and the experiment was repeated one time with the same setup.

#### Symptom development and sampling of potato plants

Plants were visually inspected daily for symptoms development: wilting, chlorosis of leaves, black rot at the stem base, aerial stem rots, haulm desiccation and/or plant death. Plants were sampled at 21 dpi by taking 1.5 cm long stem segments 5 cm above ground level and pooled per plant. The stem segments were surface-sterilized for 1 min in 70% ethanol, washed once with sterile tap water, further sterilized with 1% sodium hypochlorite (commercial bleach) for 3 min and again washed 3 times with water. Stem segments were analyzed for *D*. *solani* IPO2254 presence by plating on TSA supplemented with 150 μg ml^-1^ ampicillin and counting the GFP-positive, ampicillin-resistant bacterial colonies as described earlier (Czajkowski et al., 2014c).

### Statistical analysis

Bacterial count data were analyzed accordingly to the experimental design with a general linear model (GLM) assuming data to arise from a binominal distribution. Before applying the model, we estimated the expected counts for samples that were recorded as uncountable due to high densities of *D*. *solani*, as described previously [18]. To achieve the approximate normality, the counted data were log-transformed after adding a value of 1 to avoid taking logs of zero. The effects were considered to be significant at P = 0.05 and pair-wise differences were obtained using ANOVA. For culture tube-grown and soil-grown potato plants inoculated with bacteria the linear model adopted was a complete block design with replicates as complete blocks containing all treatments each. The visual inspection of symptom development was a dichotomous score (presence or absence). A normal distribution was assumed for plant height and weight. Data were analyzed using statistical software package Statistica ver.10 (Statsoft, www.statsoft.com).

## Results

### Characterization of features involved in viability and survival of ΦD5 in the environment

**Fig 1:**
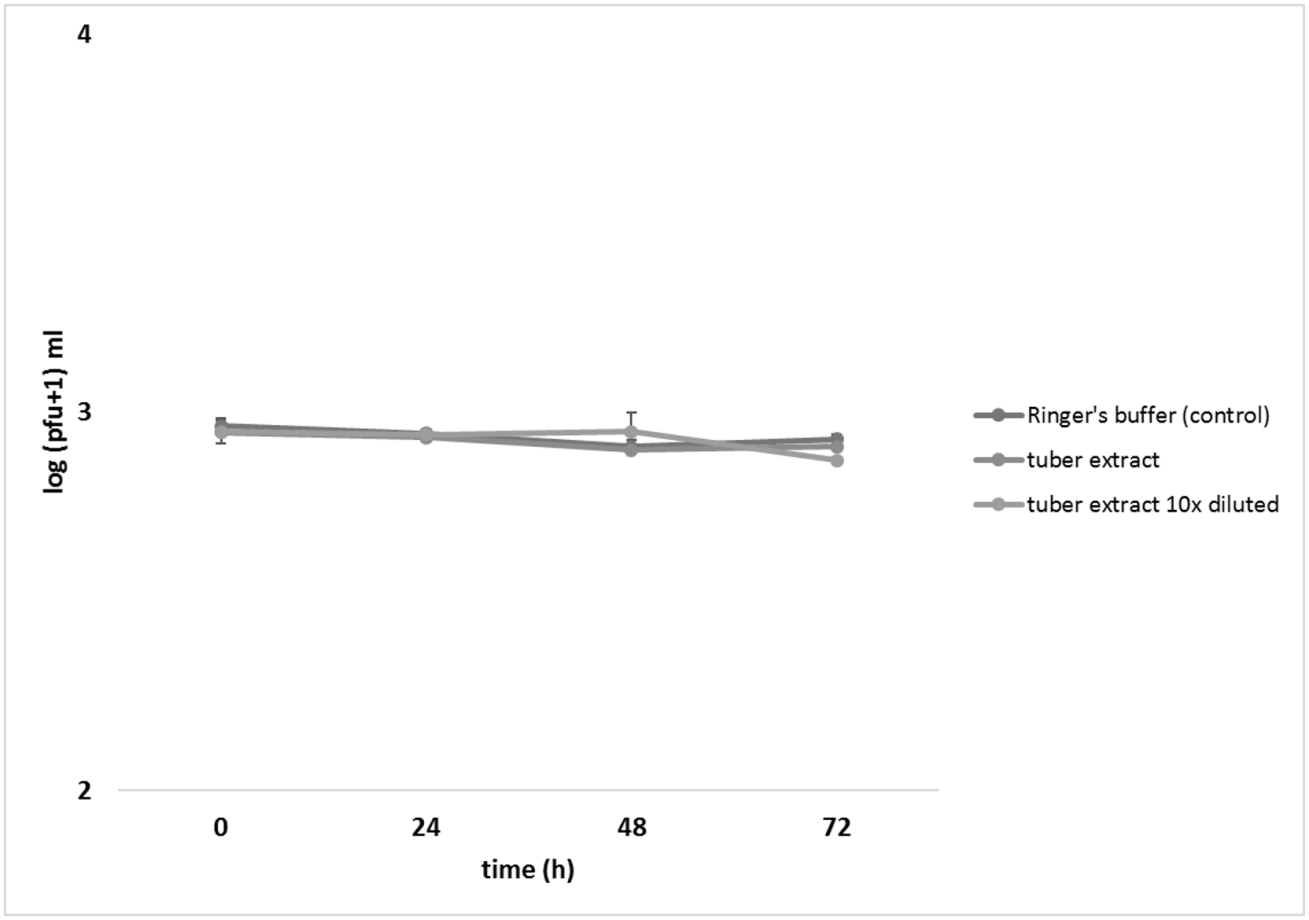
Effect of potato tuber extract on ΦD5 viability. At 0, 24, 48 and 72 h ΦD5 titers were calculated using a soft top agar assay with *D*. *solani* IPO2254 as the host. The assay was repeated independently once with the same setup and the results were averaged.

**Fig 2:**
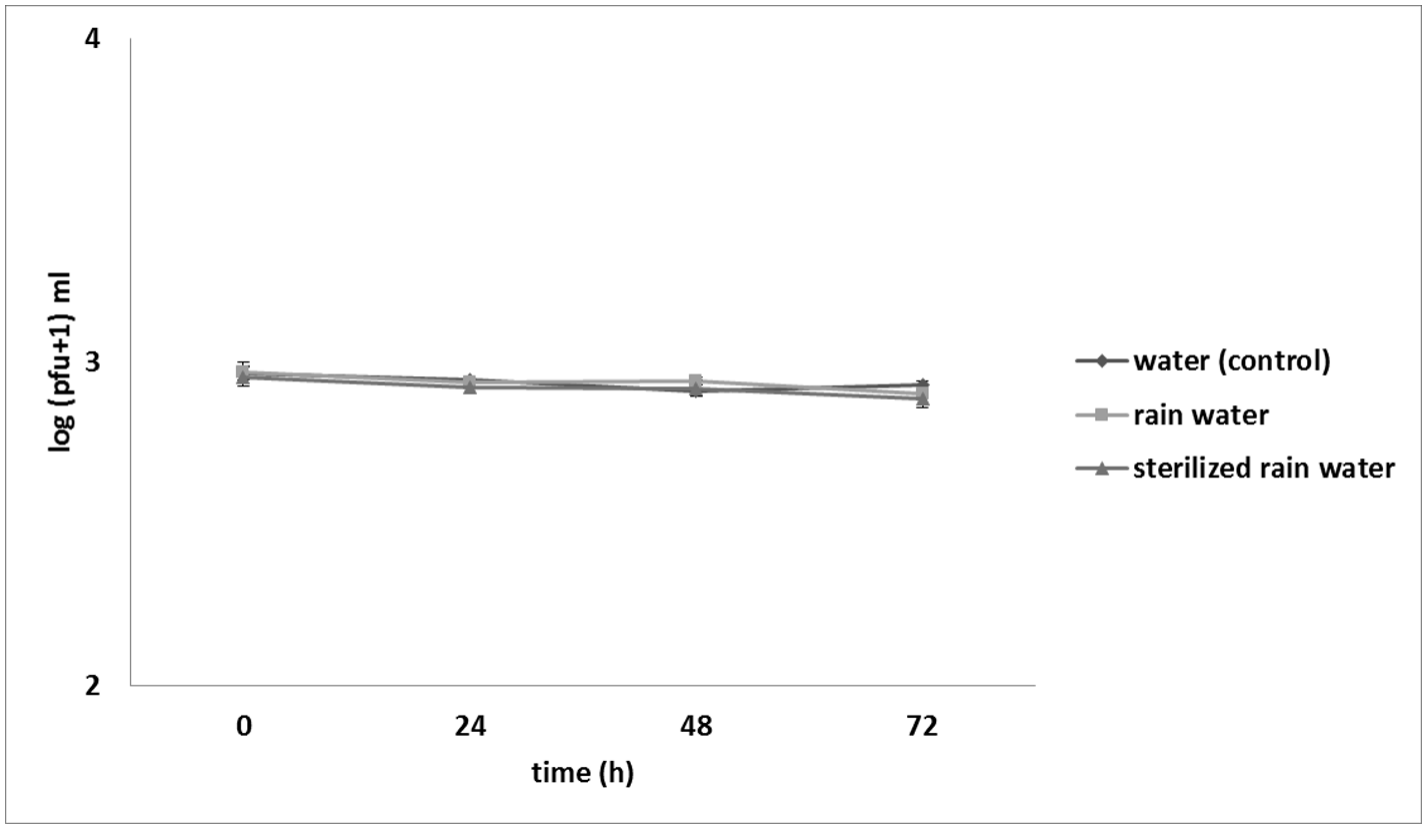
Effect of rain water on ΦD5 viability. At 0, 24, 48 and 72 h ΦD5 titers were calculated using a soft top agar assay with *D*. *solani* IPO2254 as the host. The assay was repeated independently once with the same setup and the results were averaged.

**Fig 3:**
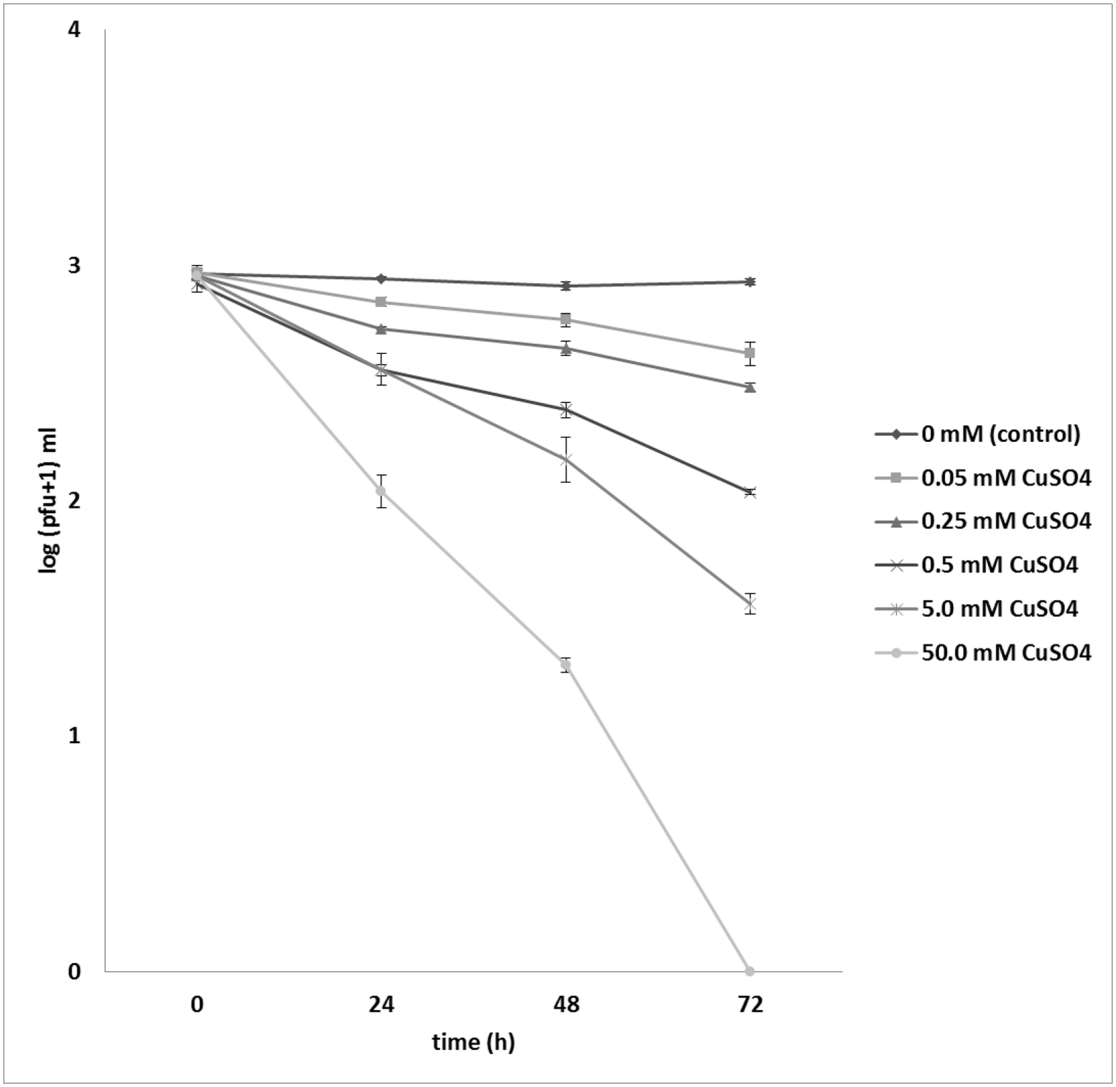
Effect of copper on ΦD5 viability. At 0, 24, 48 and 72 h ΦD5 titers were calculated using a soft top agar assay with *D*. *solani* IPO2254 as the host. The assay was repeated independently once with the same setup and the results were averaged.

Survival of ΦD5 particles in potato tuber extracts, undiluted and 10-times diluted in sterile Ringer’s buffer supplemented with an oxidant was not significantly different from that of the control over 72 h (Fig 1). Similar results were obtained when the viability of ΦD5 was tested in sterilized and unsterilized rain water (Fig 2). When the survival of ΦD5 particles in different strengths of CuSO_4_ solutions was examined, a decrease in the number of recovered phage particles was observed with increasing concentration of CuSO_4_: after 72 h, an 11% reduction of number of bacteriophage particles was observed in 0.05 mM CuSO_4_, increasing to 30% and 47% in solutions containing 0.5 and 5.0 mM CuSO_4_, respectively. In both experiments ΦD5 did not survive the 72 h incubation in 50.0 mM CuSO_4_ solution (Fig 3).

**Fig 4:**
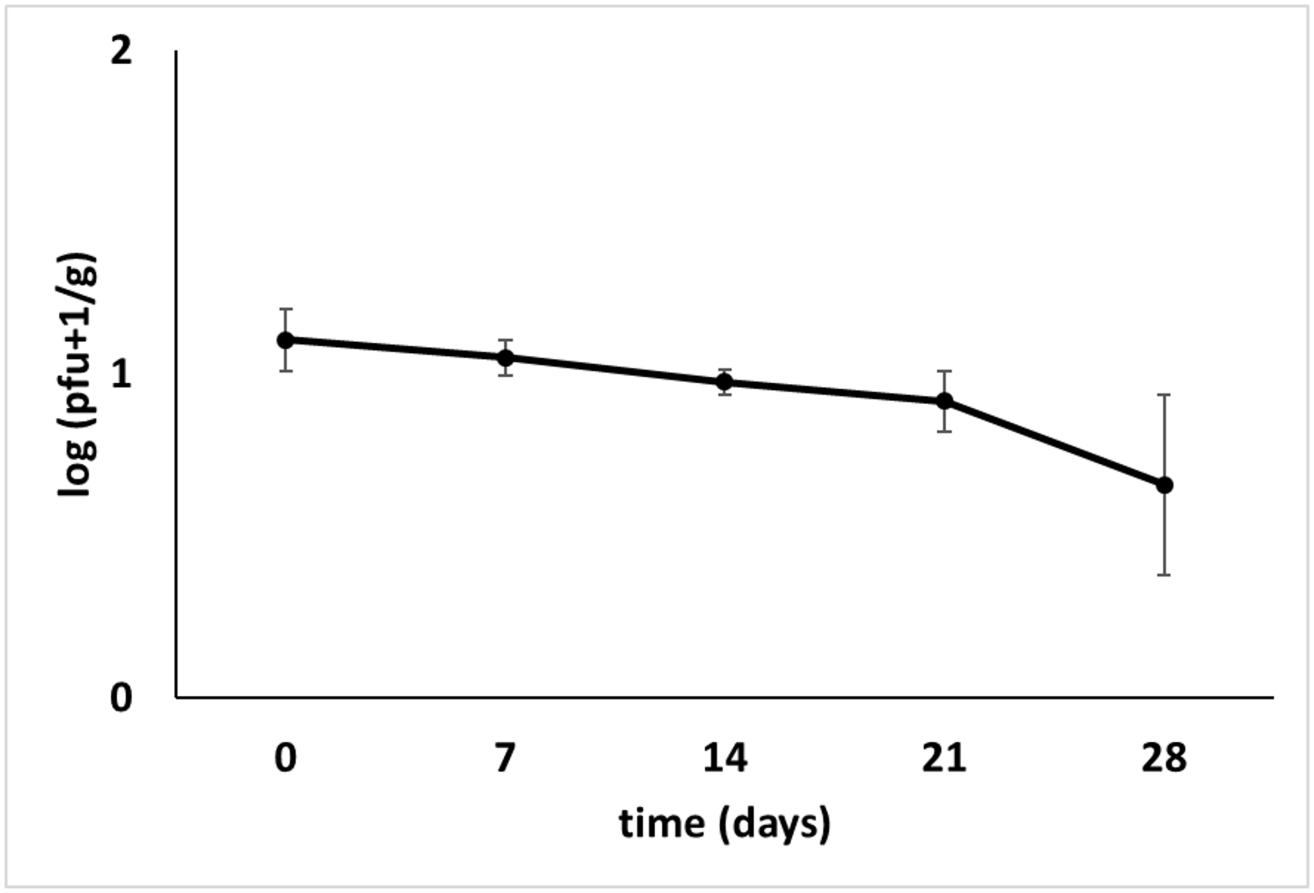
Viability of ΦD5 on the surface of potato tubers in time. Phage concentration was adjusted to 10^3^ pfu ml^-1^ in sterile Ringer’s buffer. Ten ml of phage suspension was sprayed on the surface of each tuber. For control, instead of bacteriophage suspension, sterile Ringer’s buffer was used. Tuber samples were collected at T0 (0 days), T1 (7 days), T2 (14 days), T3 (21 days) and T4 (28 days) and assayed for phage presence.

Survival of ΦD5 on the surface of potato tubers at 4–6°C and 80% RH was tested over a period of 28 days. No statistically significant reduction of number of ΦD5 particles was observed during the first 21 dpi in both experiments. On average, only a 12 and 13% reduction in bacteriophage number was observed at the final sampling time, 28 dpi (Fig 4).

**Fig 5:**
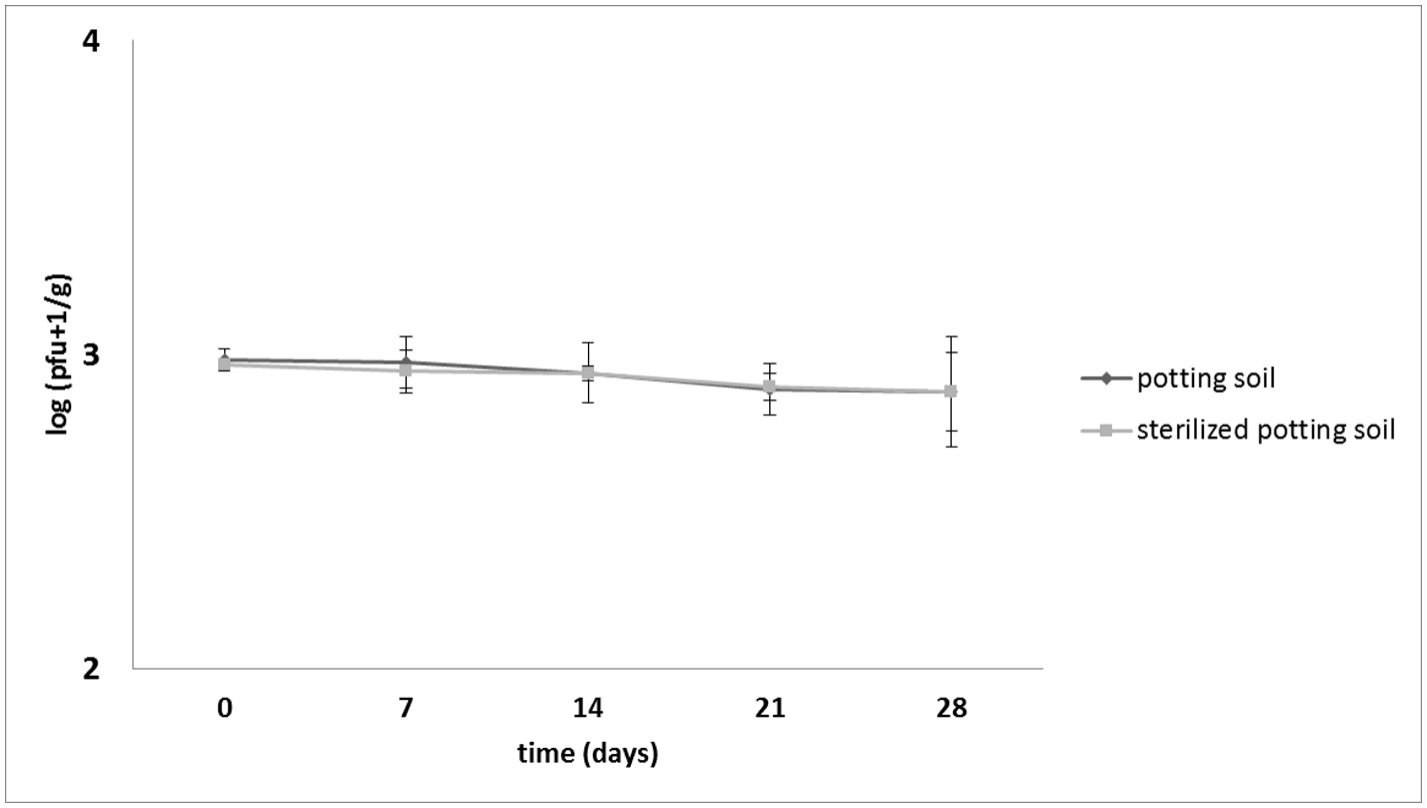
Viability of ΦD5 in potting compost. Compost samples were collected at T0 (0 days), T1 (7 days), T2 (14 days), T3 (21 days) and T4 (28 days) and assayed for the phage presence.

Survival of ΦD5 in potting compost at 10°C was tested weekly over a period of 28 days. No statistically significant difference in phage viability was observed in heat-sterilized and unsterilized potting compost in the entire course of both experiments. In both experiments, only a 3.5 and 2.8% reduction of number of ΦD5 particles were observed after 28 days in unsterilized and heat-sterilized potting compost, respectively (Fig 5).

**Fig 6:**
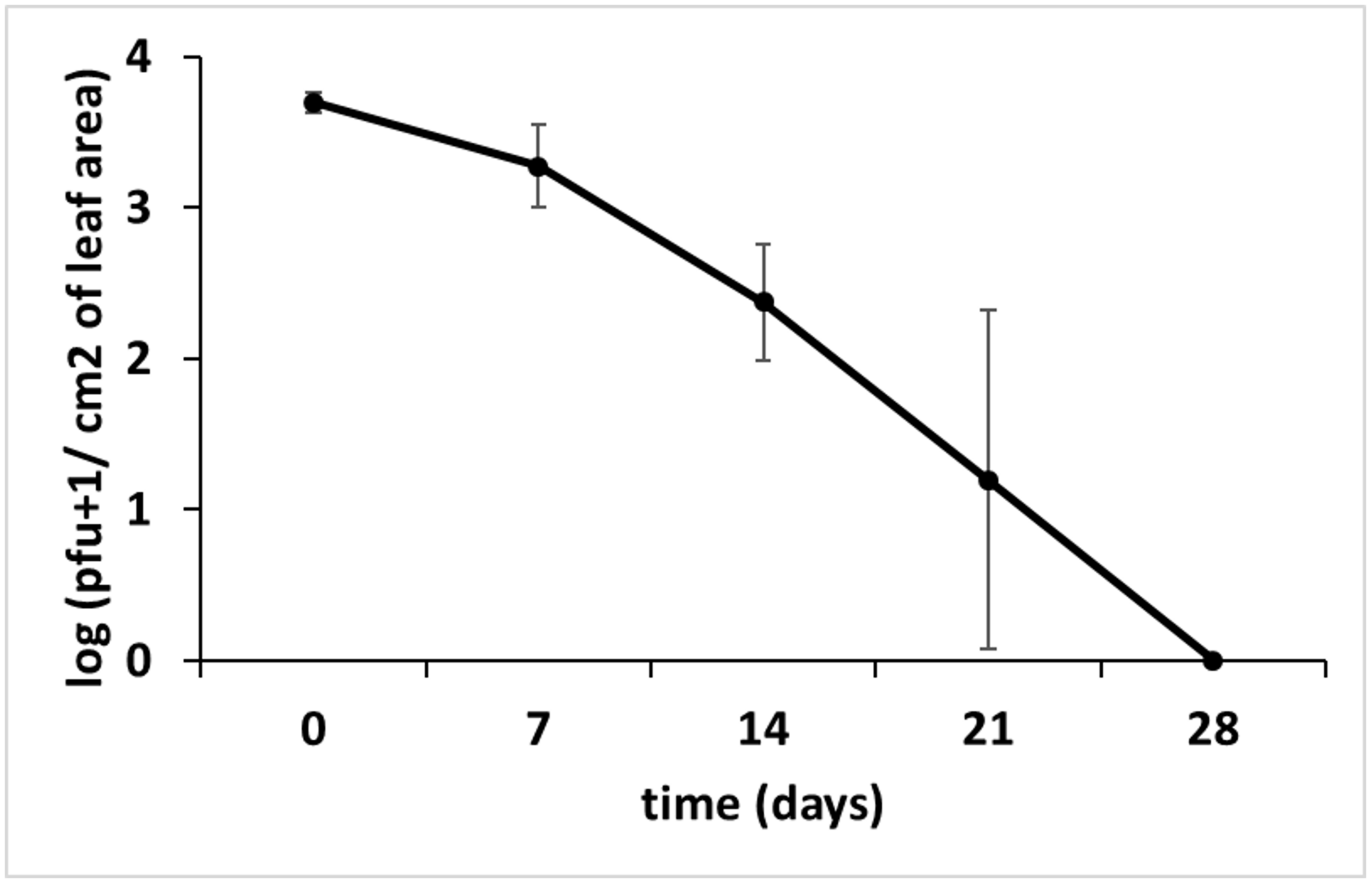
Viability of ΦD5 on the surface of potato leaves. Leaf samples were collected at T0 (0 days), T1 (7 days), T2 (14 days), T3 (21 days) and T4 (28 days) and assayed for phage presence.

Survival of ΦD5 on the adaxial surface of detached potato leaves was tested weekly over a period of 28 days. In both experiments, at 7 dpi a 13% and 15% reduction in number of bacteriophages were observed with reference to 0 dpi in experiment 1 and 2, respectively. This reduction in phage numbers increased in two repeated experiments to 34 and 58% at 14 and 21 dpi, respectively. In both experiments ΦD5 did not survive until the last sampling time, 28 dpi (Fig 6).

### Growth chamber experiments with potato plants in culture-tubes

#### Fitness of plants, disease development and *D*. *solani* populations in stems

Experiments with culture-tube grown potato plants were conducted in May (experiment 1) and June (experiment 2) 2015. In each experiment, the effect of application of ΦD5 bacteriophage on plant growth and disease development following the inoculation with *D*. *solani* strain IPO2254 was examined.

*Treatment A*. Control plants inoculated with sterile water only remained disease-free in both experiments. Plant heights in experiments 1 and 2 at 14 dpi were 4.9 cm and 4.75 cm and the average fresh weight of haulms was ca. 62 mg and 59.7 mg, respectively.

*Treatment B*. In plants inoculated with only ΦD5 bacteriophage particles, no disease symptoms developed during both experiments. Plant heights were 5.1 and 4.9 cm in experiment 1 and experiment 2, respectively, and the corresponding average fresh weight was 61 and 60 mg, respectively.

*Treatment C*. In plants co-inoculated with ΦD5 and *D*. *solani* IPO2254, the colonization of roots by the pathogen, visualized by an increase in turbidity of MS medium around the roots, was observed on four plants in experiment 1 and in three plants in experiment 2. Typical disease symptoms—blackening—were observed only on one plant in experiment 1 (5% of plants) and in one plant in experiment 2 (5% of plants). The stem rot symptoms were similar to those observed in plants inoculated with *D*. *solani* in *Treatment D* (described below). In addition, only low GFP-tagged *D*. *solani* densities (ca. 10–100 cfu g^-1^) were found in colonized plants in both experiments.

*Treatment D*. In plants inoculated only with *D*. *solani* IPO2254 (positive control) first symptoms appeared 3 days after inoculation. These included: colonization of roots and blackening of stem base close to the inoculation point. The symptoms progressed further with time in both experiments. At the end of the experiments (14 dpi), 75% of inoculated plants in both experiments showed typical blackleg symptoms and colonization of roots was observed in 100% plants in both experiments. Infection of plants with *D*. *solani* IPO2254 resulted in severe growth reduction; the average height of the highest sprout was reduced by up to 56% in experiment 1 and 27% in experiment 2. Similarly, the average fresh weight of plants was reduced by up to 62 and 50% in experiment 1 and experiment 2, respectively.

**Fig 7:**
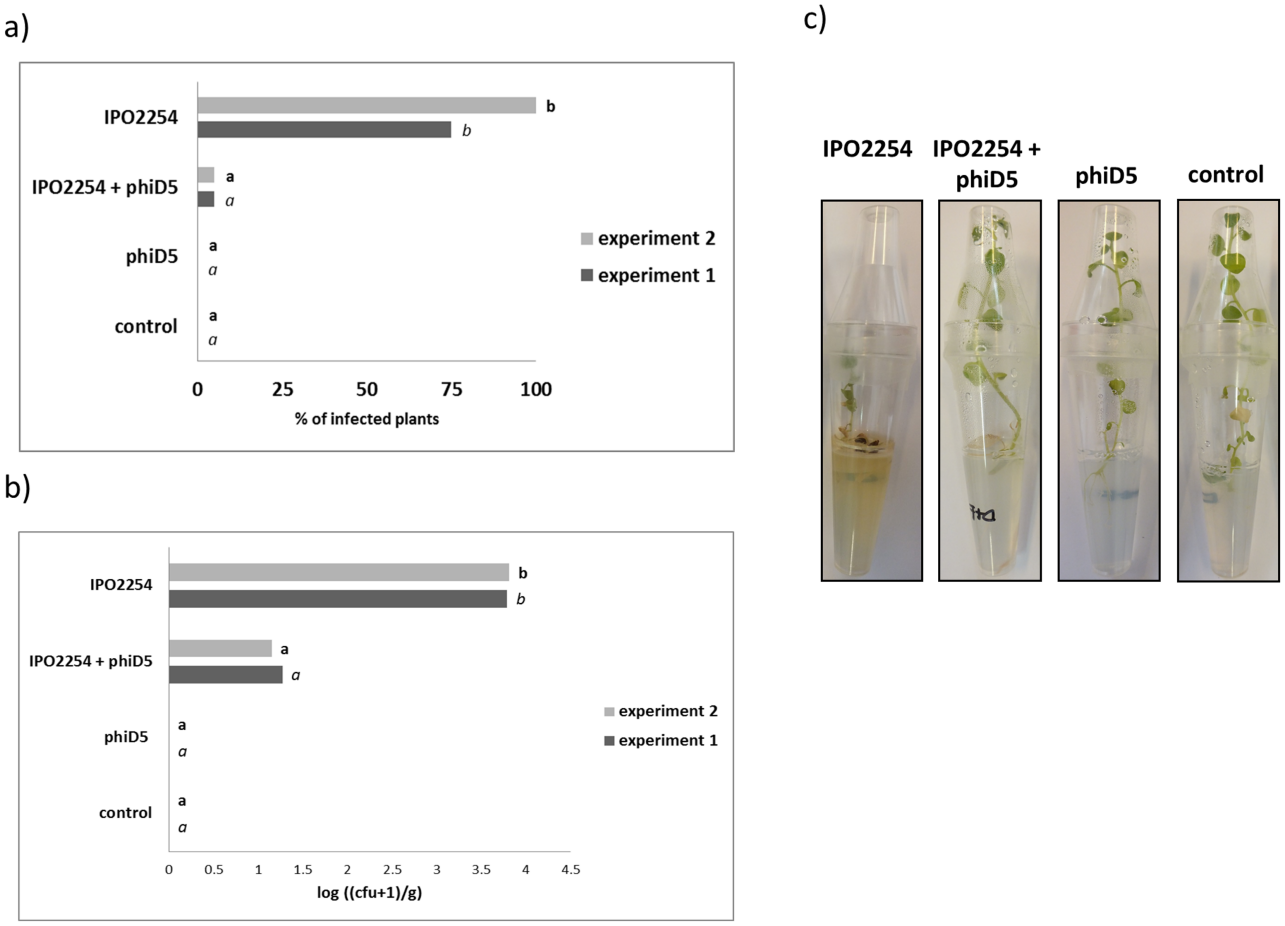
Effect of the presence of ΦD5 at 14 days on culture tube-grown plants in a growth chamber and inoculated with *D*. *solani* IPO2254. The results are presented in terms of (a) percentage of plants showing symptoms and (b) number of bacteria in stems (n = 20 plants per treatment), (c) a representative image of culture tube-grown potato plants showing disease symptoms. Values followed by identical characters are not significantly different (P = 0.005).

Fourteen days after inoculation, stem segments of culture tube-grown potato plants were collected, and bacteria extracted and plated on TSA and CVP agar plates supplemented with ampicillin to determine the percentage of infected stems and the internal bacterial population in vascular tissues of stem. No statistically significant differences in the results obtained in experiment 1 and experiment 2 were found (data not shown). In both experiments bacteria were absent in plants collected from *Treatment A* (double negative control) and *Treatment B* (plants treated with ΦD5 only). In contrast, GFP-tagged *D*. *solani* was found in all stem segments collected from plants from *Treatment* D (positive control, plants inoculated with *D*. *solani* only). In *Treatment* C (plants inoculated with *D*. *solani* IPO2254 and treated with ΦD5) only 20% (experiment 1) and 15% (experiment 2) of stem segments harbored GFP-tagged bacteria. Bacterial densities in potato stems varied widely between stems. In *Treatments C* and *D*, the average density was 10^1^–10^4^ cfu g^-1^ of *D*. *solani* strain IPO2254 in both experiments (Fig 7a and 7b).

### Growth chamber experiments with plants grown in potting compost

#### Fitness of plants

Experiments with potato plants grown in potting compost were also conducted in May (experiment 1) and June (experiment 2) 2015. In each experiment, the effect of application of bacteriophage ΦD5 on plant growth and disease development caused by *D*. *solani* strain IPO2254 in plants were examined. Plants were inspected daily for symptom development and sampled at 21 dpi.

**Treatment A1**: In control plants untreated with bacteria and bacteriophages the average stem height was 42 and 37 cm in experiment 1 and experiment 2, respectively, and the corresponding average fresh weight was 36.5 and 39 g.

**Treatment B1**: In plants grown in compost treated with ΦD5 solution, in experiments 1 and 2 the average stem heights were 43.3 cm and 40 cm and the average fresh weight was 35 g and 42 g, respectively.

**Treatment C1**: In plants grown in compost inoculated with *D*. *solani* IPO2254 and treated with bacteriophage ΦD5 suspension, the average height of the sprout was 35 and 34 cm in experiment 1 and experiment 2, respectively, and the corresponding average fresh weight of haulms was 33 and 39 g, respectively.

**Treatment D1**: In plants grown in compost inoculated with IPO2254, severe disease symptoms were present and the average heights of plants in experiment 1 and 2 were 26 cm and 27 cm and the corresponding average fresh weight was 22 g and 27 g, respectively.

#### Disease development and *D*. *solani* populations in stems

No disease symptoms were observed at any time in plants of *Treatment A1* (double negative control) and *Treatment B1* (plant grown in compost inoculated with bacteriophage ΦD5 only). In plants grown in compost inoculated only with bacteria (*Treatment D1*) no symptoms were observed in the first two weeks but they developed later starting from 14 dpi. In plants grown in compost inoculated with both bacteria and bacteriophages (*Treatment C1*), 1 plant in experiments 1 and 2, developed symptoms 21 dpi: stem blackening near the stem base and chlorosis and wilting of leaves. But 11 and 10 plants of *Treatment D1* (compost inoculated with bacteria) in experiments 1 and 2, respectively, developed more severe rotting and wilting symptoms leading to plant death.

**Fig 8:**
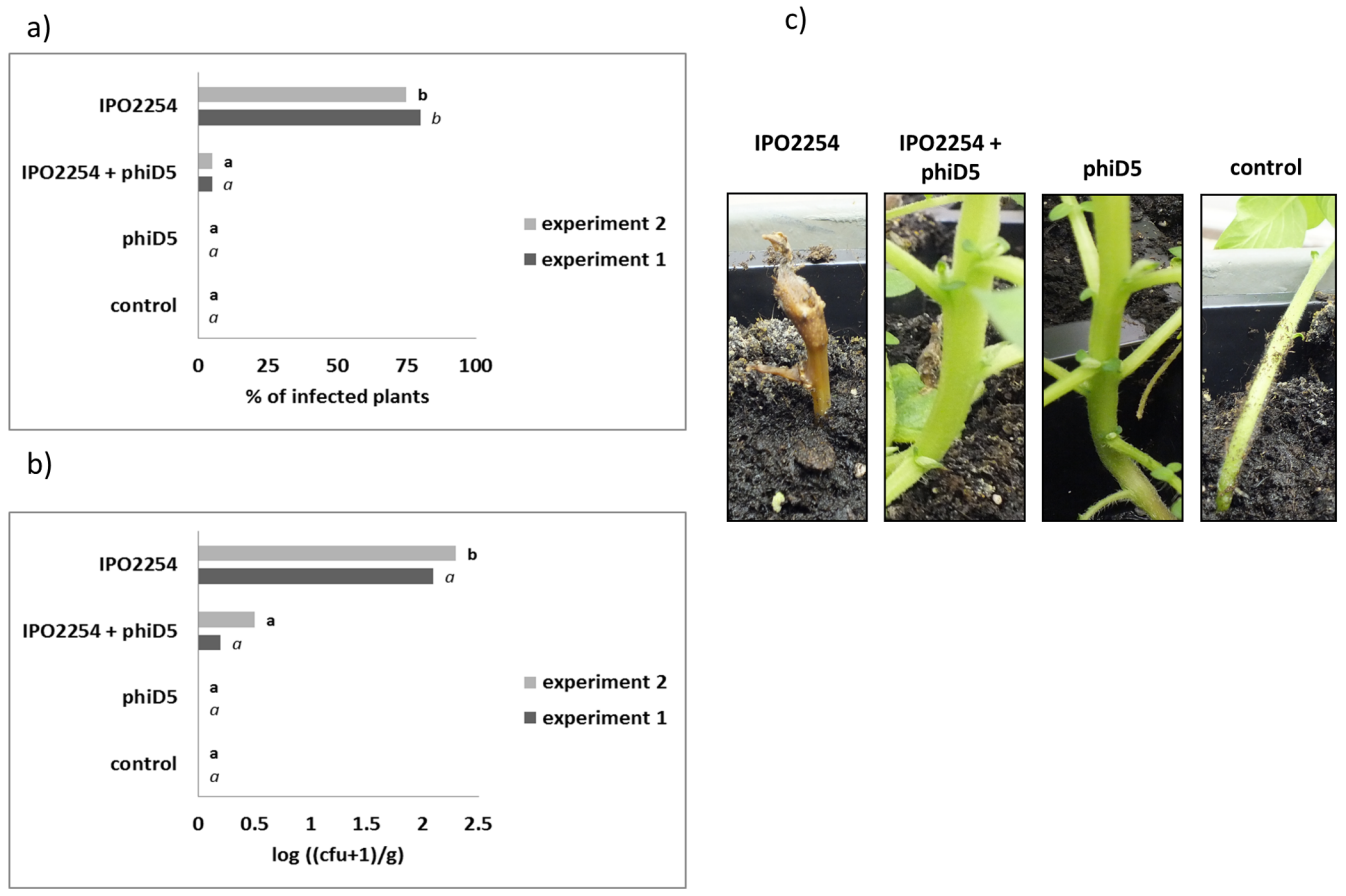
Effect of the presence of ΦD5 at 21 days on plants grown in pots in a growth chamber and inoculated with GFP-tagged *D*. *solani* IPO2254. The results are presented in terms of (a) percentage of plants showing blackleg symptoms and (b) number of bacteria in stems (n = 15 plants per treatment), (c) a representative image of pot-grown potato plants showing disease symptoms. Values followed by identical characters are not significantly different (P = 0.005).

Bacterial populations in shoots were analyzed at 21 dpi by plating stem extracts on TSA supplemented with ampicillin for selection of the GFP-tagged bacteria and by epifluorescence microscopy to assure that all bacterial colonies exhibited a GFP-positive phenotype. Densities of *D*. *solani* IPO2254 in stems in both experiments varied widely per treatment and per plant screened. No statistically significant difference in results between experiment 1 and experiment 2 were found (data not shown). In *Treatment D1* (plants grown in compost inoculated with *D*. *solani*) at 21 dpi, 10^3^–10^4^ cfu g^-1^ of stem tissue were present in both experiments. In plants from *Treatment* C1 (plants grown in compost inoculated with both, bacteria and ΦD5), at the same sampling point, ca. 10^2^ cfu g^-1^ of stem tissue were recorded. No *D*. *solani* IPO2254 was found in stems of plants in *Treatment A1* and *Treatment B1* in both experiments (Fig 8a and 8b).

## Discussion

There is very little research reported on the ecology of lytic bacteriophages of plant pathogens and on their effect on the bacterial hosts in the environment [9, 11]. To our knowledge no such studies have been performed on lytic bacteriophages infecting soft rot *Enterobacteriaceae*, specifically—*D*. *solani* hosts. This study is the first attempt to better understand the ecology of lytic bacteriophages infecting *Dickeya* spp. in the natural and agricultural environments.

In our study, unformulated ΦD5 exposed to relatively harsh environment of fresh potato tuber extract, compost, tuber surface or rain water survived for a relatively long time intact. In the phage-host interaction, the period between the release of phage particles from a lysed cell and infection of a new host cell is critical for their survival [22]. The phage particles can be exposed to environmental conditions what can lead to their physical damage resulting in a decrease in phage infection incidence [8]. Temperature, pH, humidity, sunlight radiation as well as the presence of copper ions have been shown to have a direct impact on phage viability and virulence [9]. Jonczyk and co-workers (2011) reviewed inactivation of diverse lytic bacteriophages in the environment and concluded that the viability is a multifactorial process involving several factors interacting with one another determining phage sensitivity [23]. Most cases of description of bacteriophages’ viability in the environment have come from studies concerning food production and control of decomposition-causing bacteria [24]. For example, contrasting results were obtained for phage A511: it survived for a long time on different foods such as smoked salmon, seafood, cheese, poultry meat and hot-dogs but its viability was severely restricted on fresh cabbage or lettuce [25]. Few studies have dealt with survival and viability of bacteriophages infecting phytopathogens on plants but most are related to survival in the phyllosphere and rhizosphere of different crops [26–28]. To our knowledge, no similar studies have been performed for potato plants.

In this study, ΦD5 remained viable up to 28 days on the surface of potato tubers and in potting compost, which might suggest that phage particles could be applied directly on tubers in storage and/or to field soil during planting as suggested by others [29]. Furthermore, the viability of ΦD5 on tuber surface was assessed under conditions (4–6°C and 80% RH) known to promote wound healing, hence reducing build-up of SRE populations [29, 30]. A practical implication of these results could be spraying a suspension of phage ΦD5 on tubers at the moment of planting and during or after harvest to protect potential zones of contamination, wounds and lenticels, from the risk of subsequent infection by any present SRE, as demonstrated for the control of *Streptomyces scabies* with phage ΦAS1 [31].

In contrast, ΦD5 was readily inactivated on potato leaves incubated under growth chamber conditions, with no phage surviving 28 days post application, which is in concordance with our earlier observation, that ΦD5 is sensitive to various environmental factors including desiccation and/or light inactivation [13]. Light inactivation together with desiccation are the main limiting factors affecting the viability of bacteriophages in the phyllosphere [32]. Hence, application of bacteriophages to the aboveground plant parts is ill advised [27, 33–35]. This is, however, of no great importance in the control of SRE on potato as the disease is mainly seedborne, hence any treatment needs to aim primarily at the tubers at harvest, in storage and at planting [6, 10].

In previous experiments ΦD5 significantly decreased rotting of tubers inoculated with *D*. *solani* strains IPO2222 and IFB0099 [13]. In this study, we used two different plant setups, *viz*. *in vitro* cultured potato plants and plants grown from tubers in potting compost in a growth chamber, to better characterize the effect of ΦD5 on the development of disease symptoms caused by *D*. *solani* IPO2222, as well as to study the phage effect on bacteria under more natural conditions. Treatment of media for plant growth (tissue culture and compost) with ΦD5 resulted in high level of protection against infection of the inoculated plants. This suggests that the bacteriophage might protect the potato plants from systemic infection. The degree of obtained control appears to confirm previous studies showing that the application of *D*. *solani*-specific bacteriophage LIMEstone1 to seed tubers before planting provided effective protection against blackleg in the field, as well as it significantly increased crop yield and health of progeny tubers in storage [14, 15]. Therefore, a broad-host range phage ΦD5 could be viewed at least as an equivalent control measure against soft rot and blackleg caused by *Dickeya* spp. in the field.

Several reports have shown that the application of lytic phages might be integrated successfully with other biological control measures, e.g. inducers of systemic acquired resistance, as it was shown for the management of leaf blight of onion caused by *Xanthomonas* spp. [36, 37], or antagonistic bacteria to control fire blight (*Erwinia amylovora*) of pear [38]. As copper is often the only available effective control measure against many bacterial pathogens of plants [39], its combined use with phage ΦD5 is possibly worth exploring. Copper has not been widely used against SRE in potato, except in a study where it was applied on potato leaves in field cultures to control aerial contamination of healthy plants [40]. The rationale was to protect first field-grown generation of axenically-derived potato plants from aerial borne SRE contamination which is considered as the primary source of the bacteria in seed production [40]. The results concerning the sensitivity of the phage to CuSO_4_, demonstrated in this work, showed that ΦD5 was stable in 0.05 mM solution and there was ca. 50% reduction in number of phage particles in 5 mM but they were entirely inactivated in 50 mM copper sulfate solution within 72 h at 22°C. The 5 mM copper is toxic to a wide range of microbes including plant pathogenic bacteria [39]. However, its phytotoxicity can also be a problem. Previous studies had shown that exposure of *D*. *solani* IPO2254 cultures to 2% copper sulphate (ca. 125 mM copper) resulted in bacterial death under 5 min, but this concertation was highly toxic to growing potato plants [19]. We can speculate that in the presence of bacteriophages, copper concentration could be reduced to the level at which it is no longer phytotoxic but still retains an adequate level of toxicity to SRE. A possible concentration to consider would be 5 mM. Alternatively, application of phages after or before copper treatment could be considered, as suggested previously, in case of greenhouse-grown tomato plants sprayed with copper and bacteriophages against *Xanthomonas perforans* and *X*. *axonopodis* pv. *citrumelo* [27].

In conclusion, although the results obtained in this study are promising for a biological control of blackleg and soft rot caused by *D*. *solani* with lytic bacteriophage ΦD5, there is still significant work to be done to reach commercial use. Among aspects which require further exploring before commercialization are: formulation of phages to prolong their viability/survival under different environmental conditions, optimization of application procedures, longevity of the applied (formulated) ΦD5 on tubers and in soil, and extensive field trials. Finally, it would also be useful to understand the molecular basis of infection and to characterize possible receptors on *D*. *solani* cells used by ΦD5 to attach and enter the bacterial cells, what could further lead to engineering more effective biocontrol phages.

## Ethical statement

The results presented in this manuscript did not involve any endangered or protected species, field studies, human participants, specimens or tissue samples, or vertebrate animals, embryos or tissues.
